# Solid optical tissue phantom tools based on upconverting nanoparticles for biomedical applications

**DOI:** 10.1117/1.JBO.28.3.036004

**Published:** 2023-03-11

**Authors:** Gokhan Dumlupinar, Sanathana Konugolu Venkata Sekar, Claudia Nunzia Guadagno, Jean S. Matias, Pranav Lanka, Chris K.W. Kho, Stefan Andersson-Engels

**Affiliations:** a Tyndall National Institute, Biophotonics@Tyndall, IPIC, Cork, Ireland; b University College Cork, Department of Physics, Cork, Ireland; c BioPixS Ltd. (Biophotonics Standards), Cork, Ireland

**Keywords:** phantoms, upconverting nanoparticles, standards, biophotonics, photon time-of-flight spectroscopy

## Abstract

**Significance:**

Phantoms play a critical role in the development of biophotonics techniques. There is a lack of novel phantom tools in the emerging field of upconverting nanoparticles (UCNPs) for biophotonics application. This work provides a range of UCNP-based phantom tools and a manufacturing recipe to bridge the gap and accelerate the development of UCNP-based biophotonics applications.

**Aim:**

The study aims to provide a well-characterized UCNP-based solid phantom recipe and set of phantom tools to address a wide range of UCNP-based biophotonics applications.

**Approach:**

A solid phantom recipe based on silicone matrix was developed to manufacture UCNP-based phantoms. A lab built UCNP imaging system was used to characterize upconverted fluorescence emission of phantoms for linearity, homogeneity, and long-term stability. A photon time-of-flight spectroscopy technique was used to characterize the optical properties of the phantoms.

**Results:**

In total, 24 phantoms classified into 4 types, namely homogeneous, multilayer, inclusion, and base phantoms, were manufactured. The phantoms exhibit linear behavior over the dosage range of UCNPs. The phantoms were found to be stable over a limited observed period of 4 months with a coefficient of variation of <4%. The deep tissue imaging case showed that increasing the thickness of tissue reduced the UCNP emission.

**Conclusions:**

A first-of-its-kind UCNP-based solid phantom recipe was developed, and four types of UCNP phantom tools to explore biophotonics applications were presented. The UCNP phantoms exhibited a linear behavior with dosage and were stable over time. An example case showed the potential use of the phantom for deep tissue imaging applications. With recent advance in the use of UCNPs for biophotonics, we believe our recipe and tools will play a pivotal role in the growth of the UCNPs for biophotonics applications.

## 1 Introduction

The implementation of the tissue-like synthetic models is inevitable for the development, optimization, calibration, and validation of most bio-photonics systems, especially in view of the restricted usage of human/animal tissues for ethical reasons.^1^^–^^4^ Thus, the topic of developing and using optical tissue phantoms that successfully mimic the optical properties of a human/animal tissue is significant. In general, there are three fundamental building blocks of an optical phantom—a bulk material (water, epoxy resin, agar, and silicone), a scatterer (intralipid, liposyn, milk, titanium oxide, aluminum oxide, latex, or silica microspheres), and an absorber (inks, organic dyes, hemoglobin, β-carotene, or melanin).^5^^,^^6^ A phantom can also include additional building blocks to mimic fluorescence, Raman, or other functions of interest.^1^^,^^7^ An optical tissue phantom typically comes in one of three main physical forms: liquid, semisolid (hydrogel), or solid.^8^ Although liquid phantoms are the most widely used owing to the easy, rapid, and flexible fabrication process, they suffer from a short shelf- life and most often lack having realistic tissue geometries. Unlike liquid phantoms, creating complex tissue shapes are easier by hydrogel phantoms. However, similar to liquid phantoms, their durability is short. In contrast, solid phantoms provide high durability while preserving optical properties.^9^ In addition, recent developments in three-dimensional (3D) printing technology have eased the fabrication of solid phantoms.^10^^,^^11^ Such printers enable the fabrication of complex and realistic solid phantoms in a shorter period of time. Until now, a variety of optical phantom recipes in various forms has been published with the aim to meet a broad range of needs in biophotonics, such as diffuse optical tomography, fluorescence imaging, Raman spectroscopy, and optical coherence tomography.^12^^–^^15^

In the last decade, the upconverting nanoparticles (UCNPs) have shown promises in many biomedical applications, such as super-resolution microscopy, optogenetics, deep tissue imaging, histopathology, and photodynamic therapy.^16^^–^^20^ UCNPs have an ability to undergo an anti-Stokes frequency conversion of light due to the unique energy level structures of trivalent lanthanide ions.^21^ For instance, UCNPs yield emission in shorter wavelengths in near infrared (NIR), visible, and ultraviolet regions under NIR excitation.^22^ In particular, anti-Stokes NIR-to-NIR conversion, for which both excitation and emission fall in the optical transparency window (700 to 1100 nm), generates a number of advantages. First, inside a biological specimen, deeper light penetration, as well as high signal-to-background ratio due to minimal auto-fluorescence, is plausible in that range. Moreover, UCNPs have a high resistance to photo-bleaching and do not show the photo-blinking effect under continuous irradiation; thus they are suitable for long-term repetitive imaging.^23^ However, the development of tissue mimicking phantoms in this emerging field is mainly limited to liquid phantoms with UCNPs.^18^^,^^24^^–^^26^ To the best of our knowledge, there is no recipe available in the literature to produce UCNP embedded solid phantoms for desired tissue optical properties. The availability of UCNP-based solid phantoms can help in the testing characterization and device calibration, as well as accelerate the development of UCNPs for biophotonics applications.

In this work, we present for the first time a UCNP-based solid tissue mimicking phantoms recipe and tools for biophotonics applications. UCNPs used in this study are core NaYF_4_: Yb, Tm, and Mn (CD Bioparticles, United States) with maximum absorption and emission at 976 and 800 nm, respectively. The UCNP phantom recipe is further development of a well-characterized silicone phantom recipe in the literature.^9^ We created 24 solid phantoms as potential upconverting phantom tools. The optical properties of the phantoms were characterized using photon time-of-flight spectroscopy (pTOFS). The phantoms were tested for linearity, homogeneity. and stability of UCNPs in the silicone phantom matrix. As a case study, we explored the use of UCNP for deep tissue applications with varying top layer thicknesses.

## 2 Materials and Methods

### 2.1 Phantom Recipe and Geometry

**Fig. 1:**
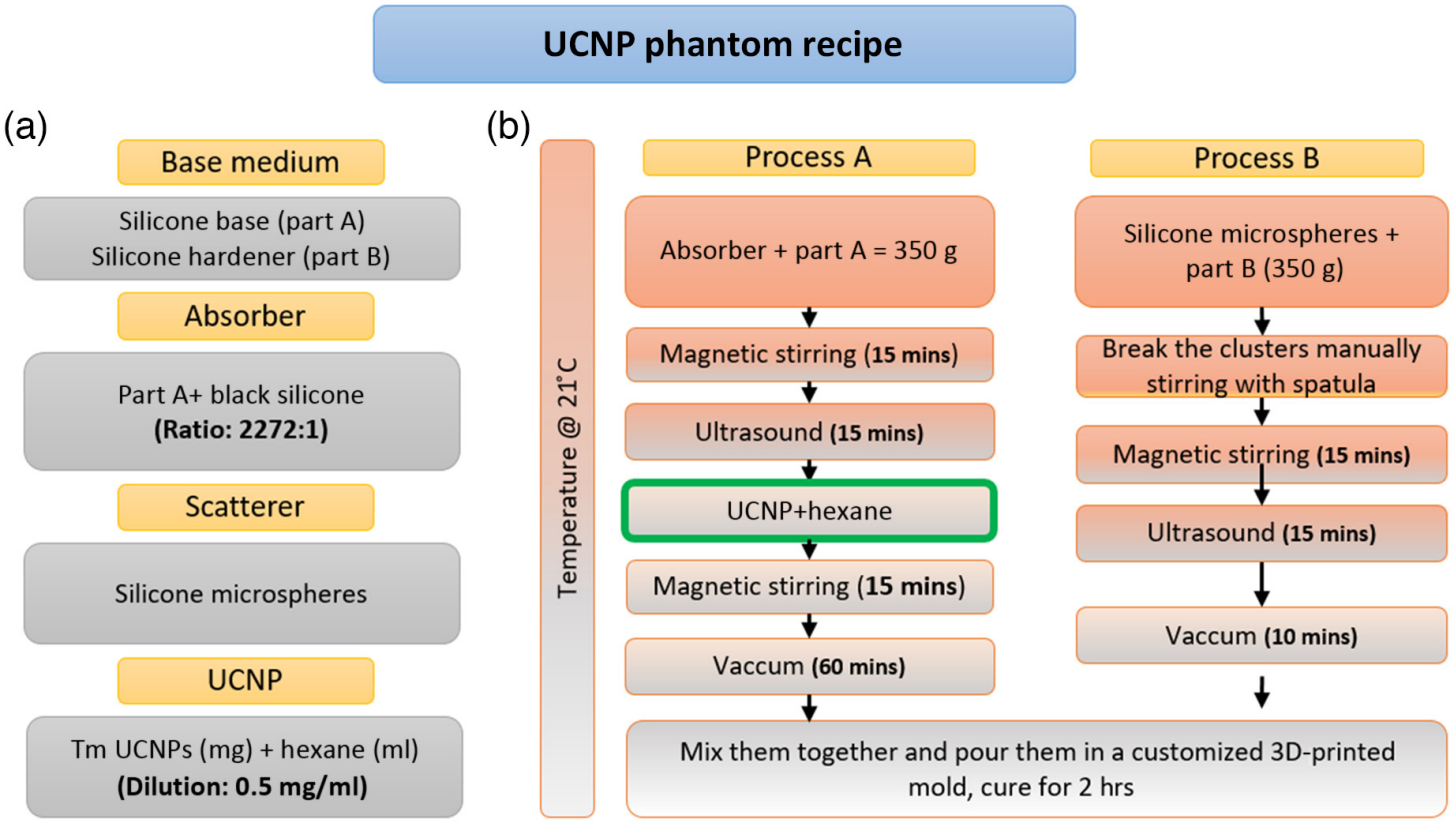
Overview of the UCNP phantom recipe: (a) components of the phantom recipe and (b) manufacturing process of the UCNP phantoms.

**Fig. 2:**
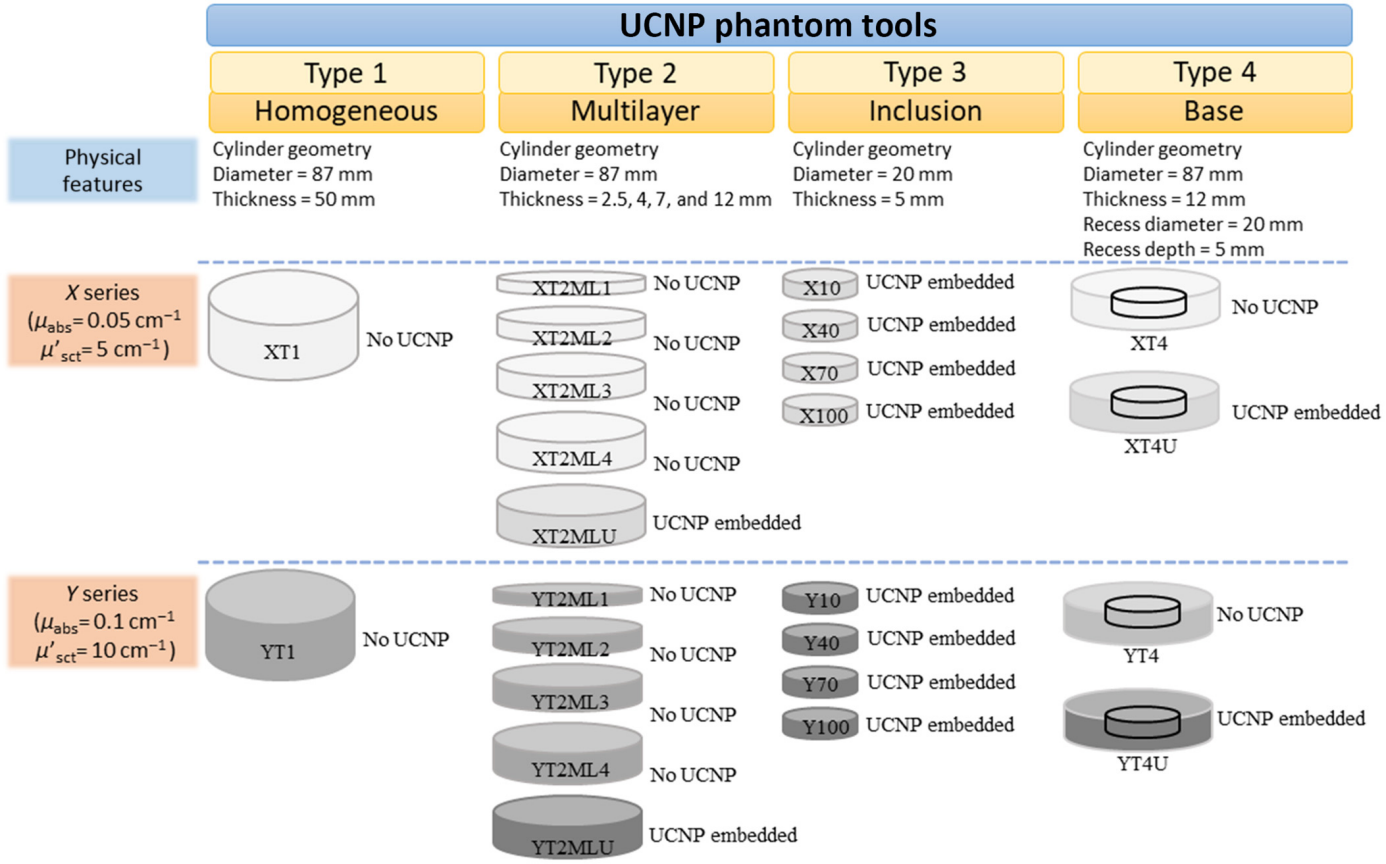
UCNP phantom tools created in this work; in total 24 phantoms, 4 homogeneous phantoms, 10 multilayer phantoms, 8 inclusion phantoms, and 4 base phantoms were created.

The detailed steps of phantom preparation are shown in Fig. 1, and the recipe follows similar steps as reported in Ref. 9. The key difference is the addition of UCNPs to part A after the ultrasound step followed by vacuum degassing for 1 hr to remove air and residual hexane from the mixture. The long vacuum degassing is critical to avoid any residual hexane in the silicone matrix, which can inhibit proper curing of the phantom mixture. In the manufacturing process, we used silicone compatible black silicone pigment (Polycraft black silicone pigment, MB Fibreglass, United Kingdom). Silica microspheres (Sigma Aldrich, United States, 440345) were used to obtain the desired reduced scattering. A schematic overview of the produced phantoms is shown in Fig. 2. In total, 24 phantoms were manufactured, divided in 2 groups of different optical properties (labeled X and Y), each group including 4 different types of solid UCNP-phantoms. All phantoms have a cylinder geometry with smooth bases and surfaces. Type 1 consists of two nonfluorescent homogeneous phantoms with a cylinder geometry of 87-mm in diameter and 50-mm in thickness. These two phantoms were made to be thick enough to characterize the recipe using pTOFS in reflectance geometry by employing a semiinfinite medium approximation.^27^^,^^28^ Type 2 is a set of five homogenous multilayer phantoms, of which only one includes UCNPs. All type 2 phantoms have the same diameter, ϕ=87  mm. However, four phantoms of type 2 have varying thicknesses (d=2.5, 4, 7, and 12 mm), and the UCNP-containing phantom of type 2 has a thickness of 7 mm. Type 3 phantoms consist of four smaller UCNP embedded solid phantoms. The type 3 inclusion phantoms have a diameter of ϕ=20  mm and thickness of d=5  mm. The final type 4 group of phantoms includes two base phantoms with and without UCNPs of thickness d=12  mm and diameter ϕ=87  mm. A recess is present in the middle of these base phantoms, with 5-mm depth and ϕ=20-mm diameter, where type 3 inclusion phantoms are inserted manually. The type 3 inclusion phantom firmly fits into the recess in the type 4 base phantom; hence, it becomes a monolithic phantom with a flat smooth top surface after the insertion.

**Fig. 3:**
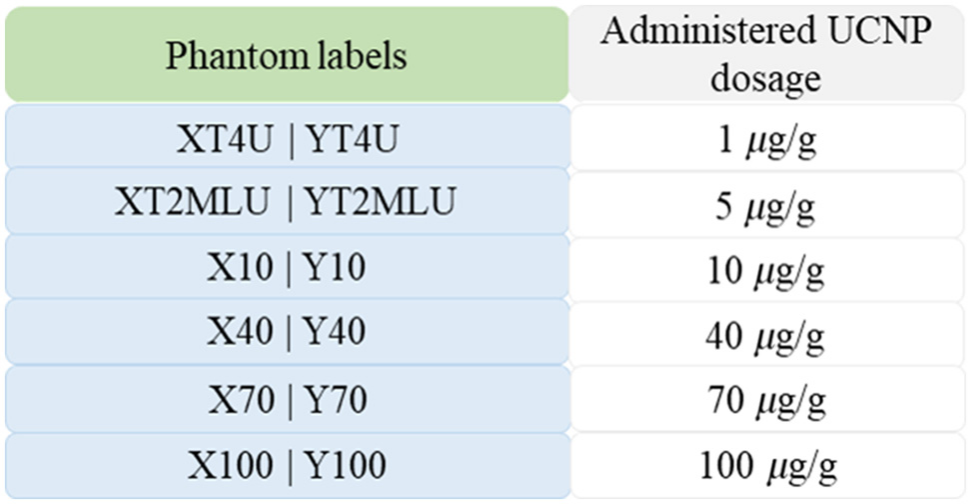
Amount of UCNPs per gram of phantom used in the formation of UCNP embedded phantoms are tabulated.

UCNP dosages used in type 3 phantoms are shown in Fig. 3. The four UCNP-containing type 3 inclusion phantoms had varying UCNP concentrations, including 10, 40, 70, and 100  μg/g. A realistic series of UCNP dosages was chosen in accordance with commonly used UCNP dosages in a number of animal studies in the literature.^18^^,^^24^^,^^29^^,^^30^

### 2.2 Instrumentation, Measurement Protocols, and Data Analysis

#### 2.2.1 pTOFS system and data analysis

A home-built pTOFS system was used for characterization of optical properties of X,Y phantoms at UCNP excitation and upconversion luminescence (UCL) wavelengths. The system is similar to the broadband system previously reported,^28^^,^^31^ which was deployed on various studies.^32^^,^^33^ The source consists of a pulsed super-continuum laser (20 MHz rep. rate, 400 to 1750 nm, SC450, Fianium, United Kingdom) and prism stage that allows for selective coupling of the chosen wavelength into a 50-μm fiber. The detection chain consists of the single photon avalanche detector (SPAD, PDM-100  μm active area, MPD, Italy) with time-to-digital converter (TDC, Picoharp 300, Picoquant, Germany) to the histogram time-of-flight of photons reemitted from the studied phantoms. The phantoms were characterized at the wavelengths corresponding to the UCL (475, 545, 660, and 800 nm) and the excitation (976 nm). pTOFS measurements were performed on type 1 homogeneous phantoms in reflectance geometry at a source detector distance of 20 mm. Signals were acquired for 1 s at a count rate of 300 kcounts/s with three repetitions. The optical properties (absorption ($\mu_{\mathrm{abs}}$) and reduced scattering ($\mu_{\mathrm{sct}}^{\prime }$) coefficients) of the X and Y phantoms were estimated by fitting the acquired photon time-of-flight distribution curves to an analytical diffusion model with extrapolated boundary conditions. A fitting range of 80% on the rising edge and 1% of the tailing edge was employed. More details on the analytical method can be found in Ref. 27.

#### 2.2.2 Phantom imaging system

The UCL intensity from tissue phantoms was measured in optical transmission geometry at room temperature. In the optical setup, all phantoms were placed at a distance of 165 mm away from the fiber tip (core 400  μm, 0.39 NA) delivering light from continuous wave (CW) excitation laser source at 975 nm (QPhotonics, QSP-975-10, United States). The incident beam diameter illuminating the front circular surface of the phantom was thereby 70 mm at the phantom location. Images of the upconverting phantoms were captured using an electron-multiplying charged coupled device (EMCCD) (Andor, iXon Ultra 897U, United Kingdom). Image J software (U.S. National Institutes of Health, United States) was used for image analysis.

During all measurements, the camera acquisition time was set to 1 s, and the laser power density was calculated to be $∼46\;{\mathrm{mW}/\mathrm{cm}}^{2}$ on the phantom surface. Furthermore, three different optical filters were used in front of the detector to effectively block excitation light that strayed in different angles. Two of them, a 794/32 nm bandpass filter and a 842-nm shortpass filter (Semrock, Models, United States), were placed inside the imaging lens system (50 mm/f1.8, Edmund Optics, United Kingdom), and the other one, a 900-nm shortpass filter (Thorlabs, FESH0900, United States), was placed between the lens system and camera sensor.

## 3 Results

**Fig. 4:**
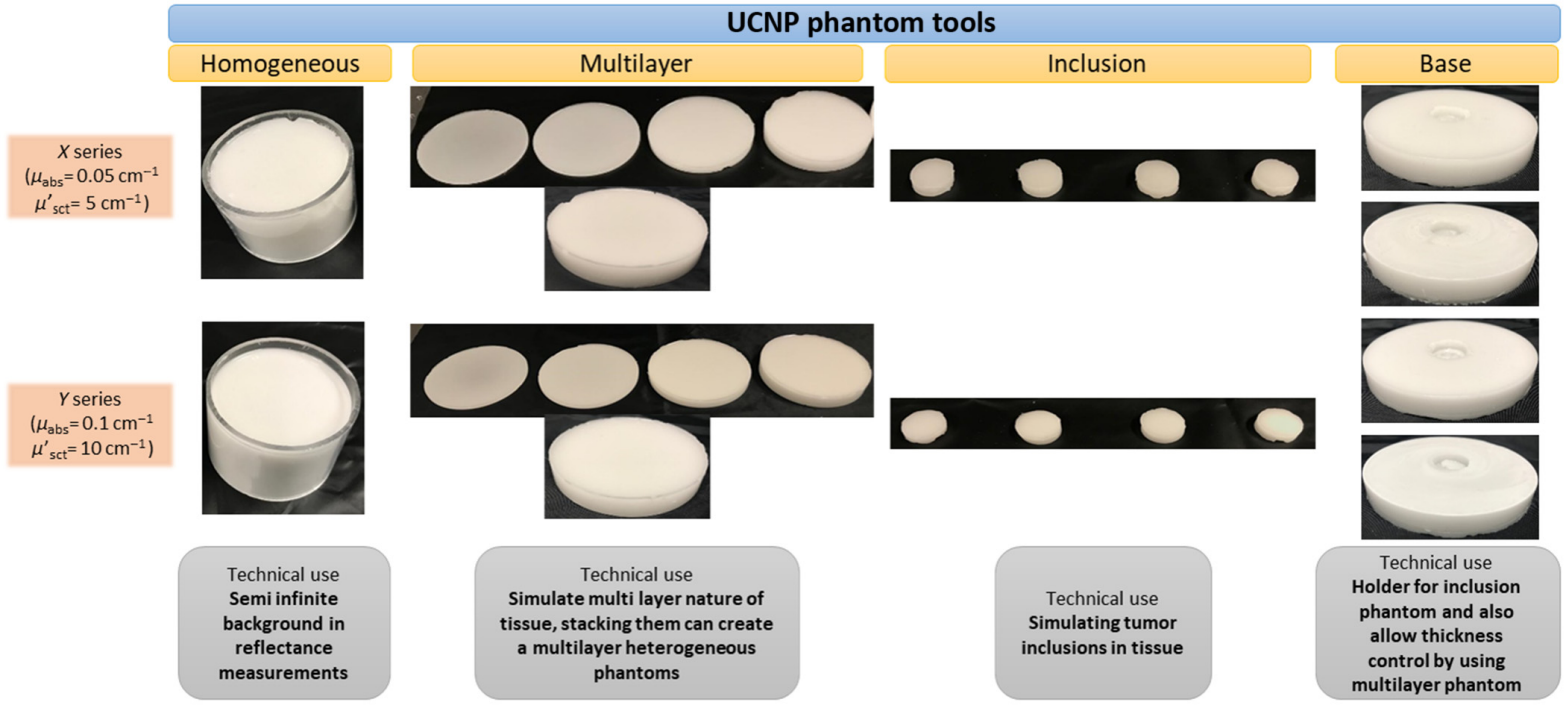
Range of phantom tools created to explore various UCNP-based applications in biophotonics; an example of technical use of each phantom type is mentioned at the bottom of the figure.

All 24 tissue-mimicking phantoms produced and studied in this project are shown in Fig. 4.

The absorption and reduced scattering characteristics of the X and Y series of type 1 homogeneous phantoms as measured with pTOFS are listed in Table 1. As expected, the absorption and scattering have similar values as reported in the literature for phantoms using the previous silicone phantom recipe for absorption and scattering phantoms.^9^ The low coefficients of variation (CVs) show that the absorption and scattering properties are independent of the UCNPs mixed into the phantoms. The phantoms are characterized at the excitation (976 nm) and the UCL lines (475, 545, 600, and 800 nm) of the UCNP used in this work.

**Table 1:** Results of optical properties of the phantoms (X, Y) used in the study. A pTOFS method is used to characterize the phantoms in reflectance geometry.

Wavelength (nm)	Absorption (${\mathrm{cm}}^{-1}$)	CV (%)	Scattering (${\mathrm{cm}}^{-1}$)	CV (%)	Phantom label code
475	0.067	2.3	6.32	1.8	X
545	0.059	2.2	5.95	1.6	X
600	0.053	0.2	5.67	0.0	X
800	0.050	0.8	5.33	1.0	X
976	0.046	2.3	5.04	1.5	X
475	0.137	1.1	12.44	1.2	Y
545	0.124	0.9	11.79	1.0	Y
600	0.112	1.4	11.13	1.4	Y
800	0.104	0.2	10.55	0.2	Y
976	0.094	0.9	9.95	0.7	Y

### 3.1 Linearity

**Fig. 5:**
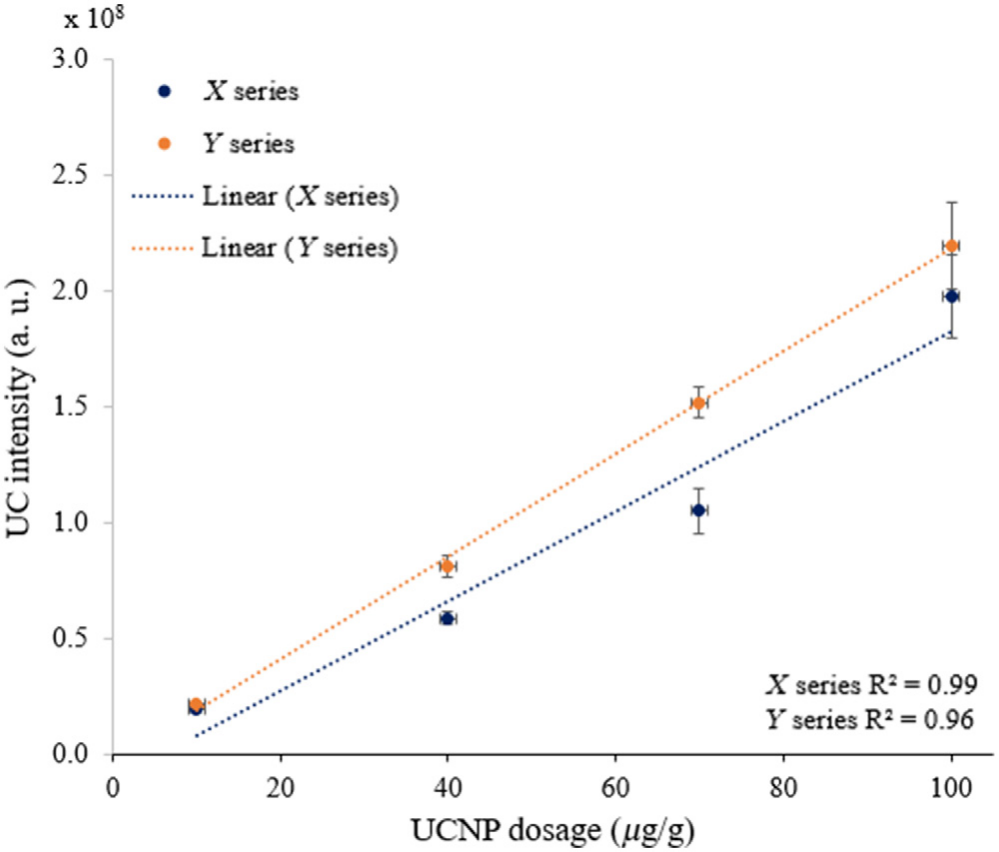
UCL intensity increases in both X and Y series of type 3 inclusion phantoms when increasing the amount of UCNPs used in phantom production: (a) X series and (b) Y series.

First, we investigated the linearity characteristics of UCL intensity with varying UCNP dosages using our home-built phantom imaging system described in Sec. 2. We used type 3 inclusion phantoms for this purpose. Each of the X and Y series comprises four UCNP embedded solid phantoms with varying UCNP dosages of 10, 40, 70, and 100  μg of UCNP per gram of phantom. We imaged each phantom in both series by an EMCCD camera (Andor, iXon Ultra 897U, United Kingdom). The total pixel value of each phantom image was extracted by Image J software (U.S. National Institutes of Health, United States) after the background correction of ambient light. In Fig. 5, we present the total pixel value as UC intensity and show its changing trend over varying UCNP dosages. It is clear that increasing the amount of UCNPs in the phantom yields a higher UCL intensity.

**Fig. 6:**
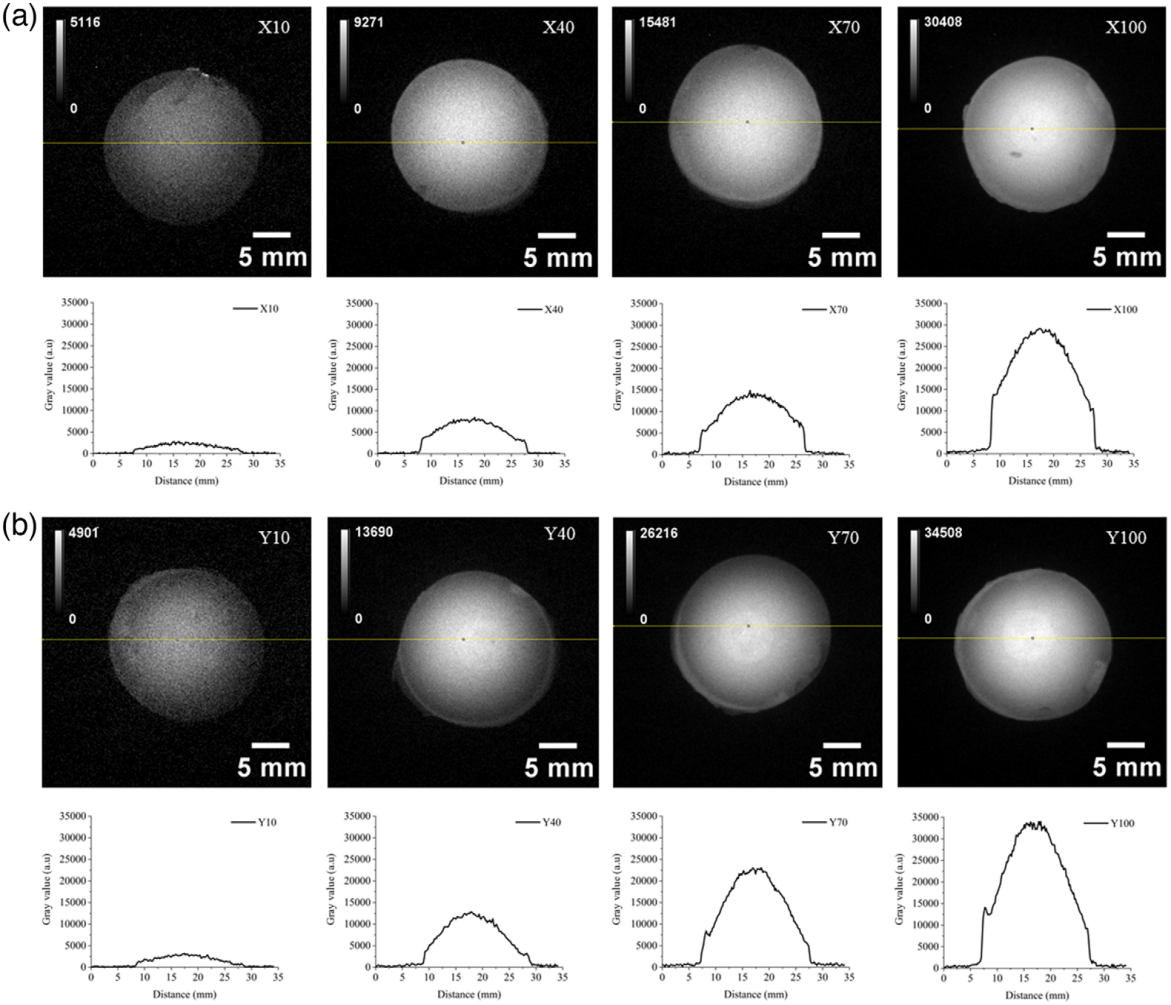
Inclusion phantoms of X and Y series show higher UCL when the UCNP dosage used in the phantoms is increased: (a) X series and (b) Y series.

We present the image of X and Y series with corresponding UCL intensity, under 976 nm excitation (power desity is $∼46\;{\mathrm{mW}/\mathrm{cm}}^{2}$) in Fig. 6. The intensity profiles show a reasonably uniform distribution of the UCL intensity over the phantoms.

### 3.2 Stability

**Fig. 7:**
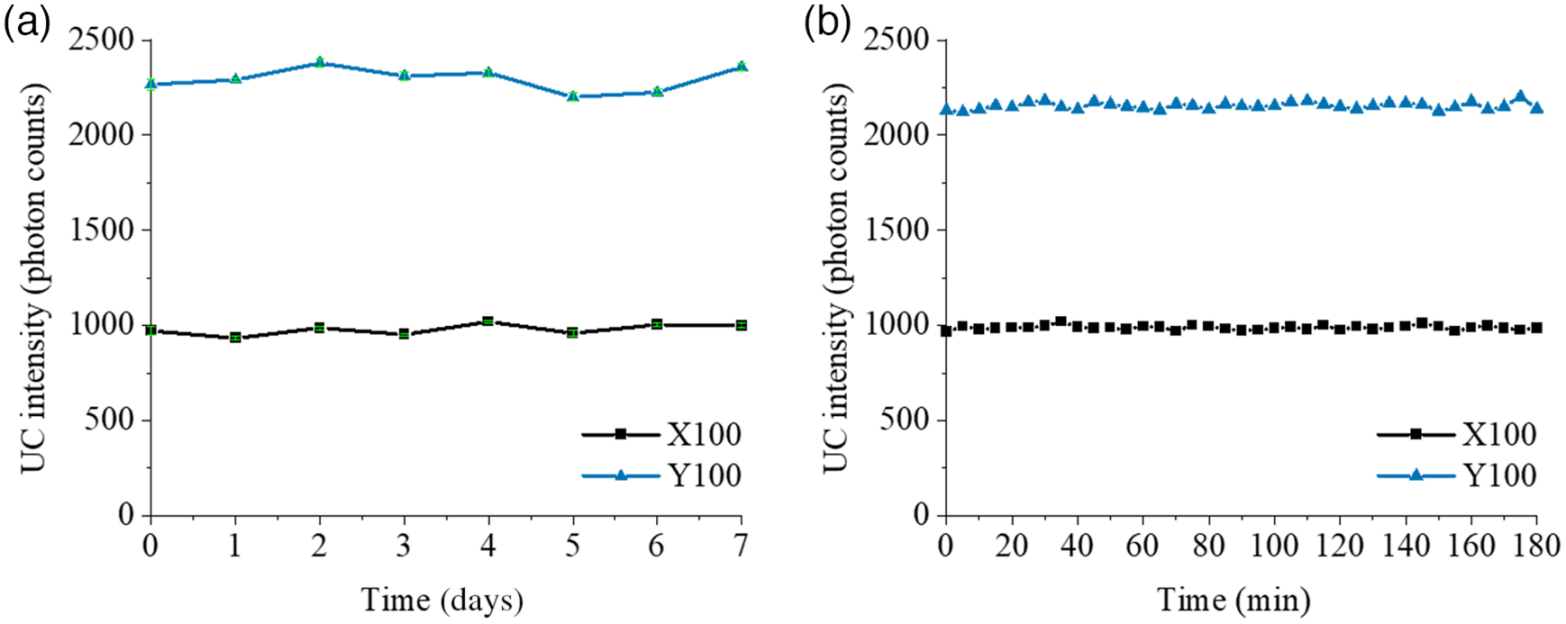
Photostability of UCNP-based solid phantom labeled as X100 and Y100: (a) frequent use scenario and (b) continuous long time irradiation scenario.

Photostability is a significant factor that contributes the reliability of an optical phantom. For this purpose, we investigated the photostability of our UCNP-based phantoms (X100 and Y100) in two different scenarios. First, we studied the behavior of the UC intensity under frequent use of these phantoms over a relatively long period of time. The UC intensity at 800 nm of the phantoms was measured by a spectrometer (Qepro, Ocean Insight, United States) under excitation of 976-nm CW laser light (power density $\approx 2.3\;{W/\mathrm{cm}}^{2}$) over 8 days. The UC intensity trend through 8 days is presented in Fig. 7(a), where each data point is an average of seven measurements recorded in each day. The time difference between two consecutive measurements out of seven is set to be 1 hour. Through 8 days, the CV of the UC intensity is calculated to be 2.9% and 2.7% for X100 and Y100, respectively. This shows that UC-based X100 and Y100 solid phantoms are highly photostable under frequent use.

In second scenario, we investigated how stable the UC capable optical phantoms are under continuous irradiation over 3 hrs. We measured the UC intensity of the X100 and Y100 phantoms every 5 min through 180 min under continuous excitation of 976-nm CW laser light, where we kept the power density at approximately $2.3\;W/{\mathrm{cm}}^{2}$. The UC intensity behavior of these phantoms under a long light exposure time is presented in Fig. 7(b). We calculated the CV of the UC intensity to be only 1% for both phantoms. This shows that X100 and Y100 phantoms do not show any photobleaching effect after a 180 min exposure of 976 nm of CW laser light.

In between measurements, the phantoms were stored in a dark box at room temperature and recordings were conducted once a month to understand the long-term stability of the phantom. The stability of UCNP emission was found to be <4% in the CV over 4 months. We believe the phantoms can stable much longer as the UCNP are shown to be photostable ^23^ or do not react with the silicone matrix. The long-term stability of the phantom is critical, particularly in applications such as UCNP phantom reference standard for calibration of biophotonics systems.

### 3.3 Case Study for Deep Tissue Imaging

**Fig. 8:**
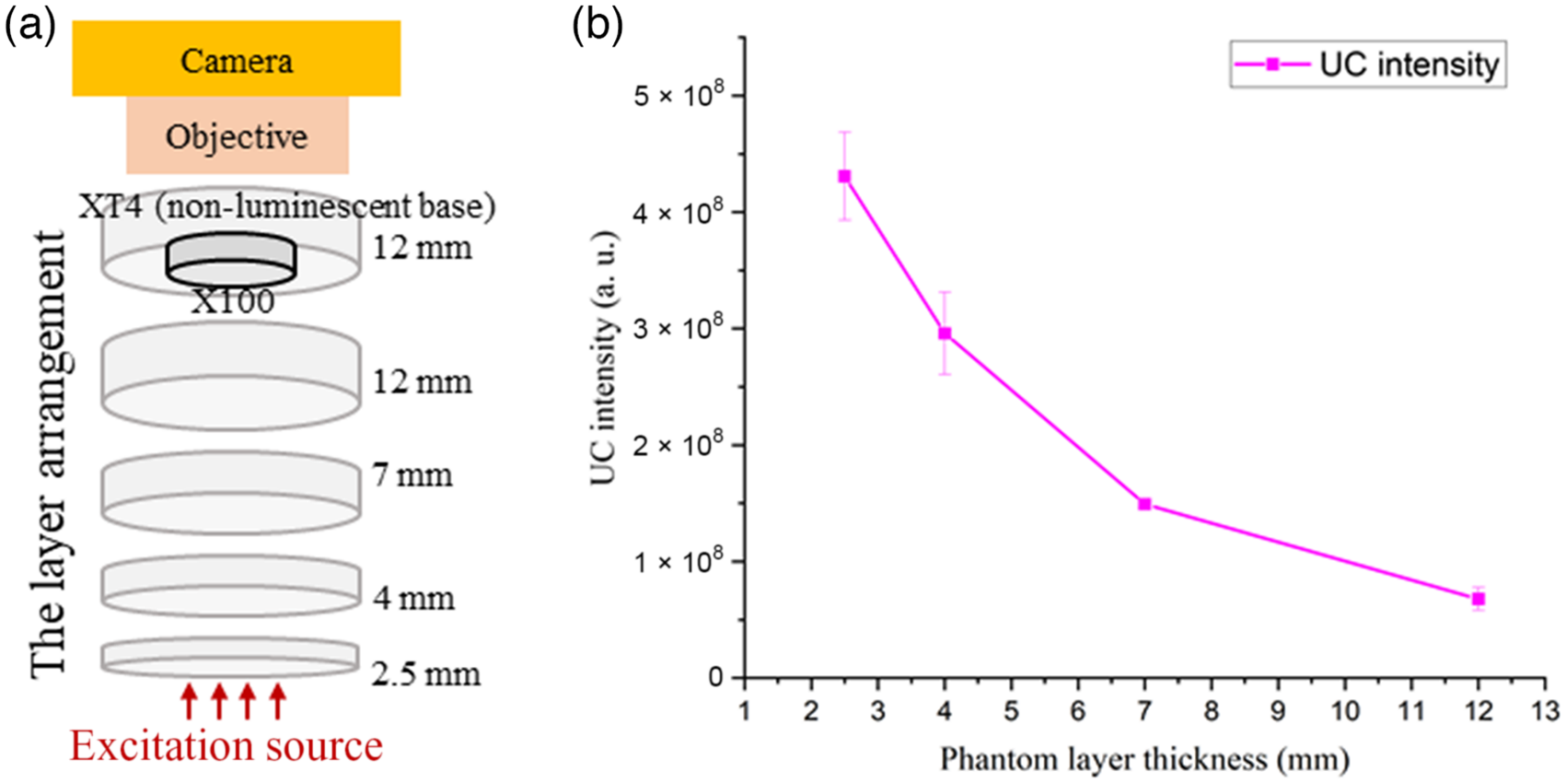
(a) Multilayer deep tissue UCNP inclusion phantom arrangement to simulate UCNP luminescence from different depth of tissue. (b) The resulting luminescence intensity as a function of layer thickness.

The optical phantoms are important tissue mimicking tools for biomedical applications. Hence, we utilized our UCNP-based solid phantoms as a potential tissue-simulating tool in deep tissue imaging in the NIR region. We measured the UCL intensity of the X100 UCNP featuring phantom at four depths by inserting multilayer phantoms of varying thicknesses. The schematic of the multilayer phantom layer arrangement for this study is depicted in Fig. 8(a).

**Fig. 9:**
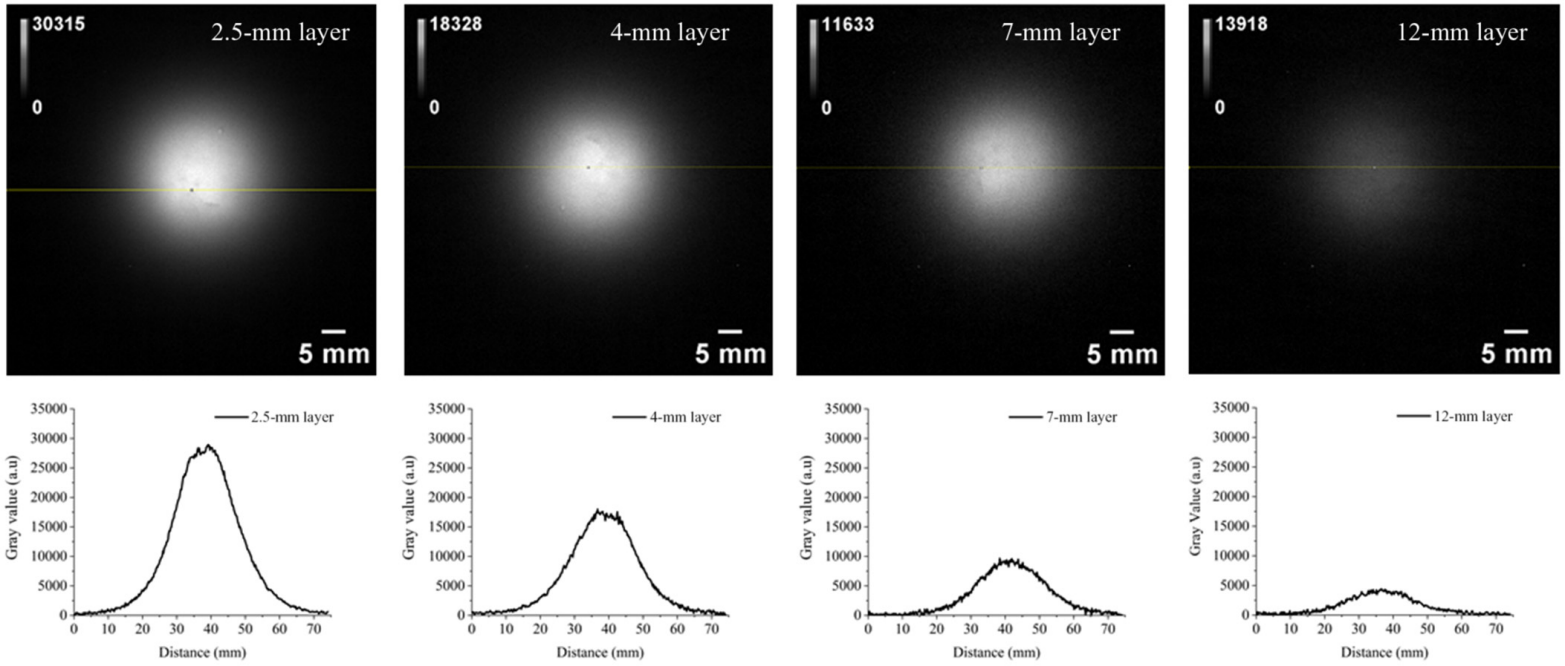
Attenuation strength of UCL from the X100 inclusion phantom changes when varying the phantom layer thickness including 2.5, 4, 7, and 12 mm.

Initially, the X100 upconverting inclusion phantom, as an example, was embedded into the designated recess in the type 4 nonluminescent solid base phantom. X100 stays firmly inside the recess, and the top surfaces align well; hence, it looks like a one-piece phantom. Then, type 2 multilayer phantoms with the four thicknesses were placed below to simulate different tissue thicknesses as shown in Fig. 8(a). No air gap is formed between the layers because the surface of the X100 inserted top phantom, as well as the surfaces of all multilayer phantoms, is smooth. Hence, they are always in contact as if they are a single piece phantom in appearance. We investigated the UCL intensity variation while changing the thickness of the phantom layer. The UCL intensity of X100 decreased when a thicker phantom layer was added, as shown in Fig. 8(b). As expected, UCL was attenuated in the presence of a thicker phantom layer. Less excitation light penetrates with increasing the phantom layer placed before the X100 phantom. The change in UCL is seen in the phantom images shown in Fig. 9. The addition of a thicker layer increases the diffusion of phantoms, which results in a low UCL intensity and lower resolution of the inclusion phantom placed under thicker multilayer phantoms. This exploration demonstrates one example of a phantom to understand and quantify the loss of signal strength and resolution of UCL in deep tissue imaging.

## 4 Discussion

Four types of UCNP-based tissue phantoms were manufactured in this work. We divided them into four categories based on the geometry, shape, size and technical use. Type 1 is a homogeneous bulk phantoms; the primary use of these phantom is to simulate semiinfinite medium in reflectance geometry. This phantom can be used as a reflectance standard or to simulate human or animal tissue in reflectance geometry. In this work, we used type 1 phantoms to characterize the optical properties (absorption and reduced scattering) of the X and Y phantoms. Type 2 is the multilayer phantoms that can simulate the heterogeneous nature of biological tissues. Using an appropriate mold structure, our recipe can be used to accurately mimic tissues having a multilayer structure such as human skin. Type 3 phantoms are simply inclusion phantoms; they can be used to simulate the tumor tissue of different geometries and sizes. The uptake of the UCNPs in the tumor can be simulated by varying dosages of UCNPs in the phantom. Type 4 phantoms are inclusion base phantoms. As the name suggests, they are the hosting platform for the type 3 inclusion phantoms, and they provide perfect matching of size and geometry, thus simulating the boundary and surrounding of tumor tissue.

From Table 1, we see that the scattering of phantom decreases with increasing the wavelength. This is expected as predicted for phantoms dominated by Mie scattering.^9^ The absorption decreases as well with increasing the wavelength, which is the characteristic of the carbon black used in the phantom as the background absorbing material. The optical properties of the phantoms were chosen to mimic typical optical properties of human tissue in the range of 690 to 910 nm, the phantom Y representing human muscle ($\mu_{\mathrm{abs}}=0.1\;{\mathrm{cm}}^{-1}$ and $\mu_{\mathrm{sct}}^{\prime }=10\;{\mathrm{cm}}^{-1}$), whereas phantom X represents low absorption seen in abdominal tissues ($\mu_{\mathrm{abs}}=0.05\;{\mathrm{cm}}^{-1}$ and $\mu_{\mathrm{sct}}^{\prime }=5\;{\mathrm{cm}}^{-1}$).^34^ A well-defined base recipe model was used to define the concentrations of the absorber and scatterer to achieve these target optical properties.^9^ From Fig. 5, it is clear that the recorded UCL intensities of the X series of inclusion phantoms are lower than that of Y series of inclusion phantoms at all four UCNP dosages. This is expected as the phantoms of X series have less scattering than that of Y series ($\mu_{\mathrm{sct}}^{\prime }=10\;{\mathrm{cm}}^{-1}$ versus $\mu_{\mathrm{sct}}^{\prime }=5\;{\mathrm{cm}}^{-1}$). A high amount of scattering (high-reduced scattering) allows for a longer photon path length inside the phantom, which results in a higher UCL intensity. The linear fit in Fig. 5 shows deviation from the expected value; this can be related to errors across multiple measurements, camera noise (error bars), and loss of some phantom material while unmolding the type 3 inclusion phantom from the phantom mold. The boundary of the inclusion phantoms is less luminescent as compared with the middle of the phantom, as shown in Fig. 6. This can be related to two effects: (1) some of the photons emitted close to the boundary are lost to the surrounding area via photon scattering across the sidewall. These photons are not captured by the imaging system. As scattering across the sidewall is more likely the closer to the wall they are, it will result in a gradient in the measured intensity in the recorded images close to the edge of the phantom. (2) The 20-mm (diameter) type 3 inclusion phantom was supported by a 3D-printed holder with 15-mm-diameter aperture, which adds further to the darkness of the boundary (see Fig. 6). This gradient is not seen in Fig. 9 (most clearly seen for the 2.5-mm phantom thickness), where the inclusion phantom is surrounded by a so-called base phantom with the same scattering and absorption properties. The few bright spots seen in the images can be related to the presence of UCNP clusters. Such clusters were also present in the hexane solution added to the phantom recipe (not shown). The use of the UCNP hexane mother solution with no clusters can solve this issue. It is important to note that the UCNP clusters are distributed uniformly across the phantom; this will make the luminescence intensity uniform for macroscopic applications such as the deep tissue imaging system as shown in Fig. 8(b).

## 5 Conclusions

A first-of-its-kind UCNP-based solid phantom recipe was developed, and four types of UCNP phantom tools to explore biophotonics applications were presented. In total, 24 phantoms classified into 4 types, namely homogeneous, multilayer, inclusion, and base phantoms, were manufactured. X and Y series of UCNP embedded phantoms exhibited relatively linear behavior over the dosage range of UCNPs. The phantoms were found to be stable over a limited observed period of 4 months with a CV of <4%. They also showed excellent photostability under the frequent use and long continuous irradiation with moderate power density. The deep tissue imaging case showed that increasing the thickness of tissue reduced the UCNP emission. An example case showed the potential use of the phantom for deep tissue imaging applications. With recent advances in the use of UCNPs for biophotonics, we believe that our recipe and tools will play a pivot role in the growth of UCNPs for biophotonics applications.
